# The California adverse childhood experiences screening roll-out: a survey study of ACEs screening implementation in primary care

**DOI:** 10.3389/fpubh.2025.1446555

**Published:** 2025-04-02

**Authors:** Clare Viglione, Kathleen Soon, Sandra Wittleder, Kyung E. Rhee, Renée Boynton-Jarrett, Pradeep Gidwani, Blanca Melendrez, Eric Hekler

**Affiliations:** ^1^Herbert Wertheim School of Public Health and Human Longevity Science, University of California San Diego, San Diego, CA, United States; ^2^Design Lab, University of California San Diego, San Diego, CA, United States; ^3^Division of Health Services Research, Department of Pediatrics, Boston Medical Center, Boston, MA, United States; ^4^Department of Medicine, NYU Grossman School of Medicine, New York, NY, United States; ^5^Department of Pediatrics, UC San Diego School of Medicine, San Diego, CA, United States; ^6^American Academy of Pediatrics, California Chapter 3, San Diego, CA, United States; ^7^UC San Diego Altman Clinical and Translational Research Institute Center for Community Health, University of San Diego, San Diego, CA, United States

**Keywords:** adverse childhood experiences, financial incentive, implementation evaluation, primary care, child health and development, policy, provider behavior, screening tools

## Abstract

**Background:**

California adopted universal screening of adverse childhood experiences (ACEs) in January 2020 and dedicated significant financial and human resources to “ACES Aware,” a statewide campaign to scale ACEs screening throughout the state. Provider perspectives after the roll-out of ACEs Aware have been understudied. The aim of this study was to understand provider perspectives on universal ACEs screening in primary care. We explored indicators of acceptability, utility, and barriers and facilitators of screening for ACEs. We also investigated treatments offered for disclosed ACEs.

**Methods:**

A cross-sectional survey with quantitative and qualitative components was distributed via Facebook, Twitter, and electronic listservs between March and April 2022, 2 years after the launch of ACEs Aware. The survey included the validated and reliable “Acceptability of Implementation Measure” and “Feasibility of Implementation Measure” as well as multiple choice, ranking, and free-text items to understand determinants of screening and treatment approaches.

**Results:**

Eighty two primary care providers in California, working primarily in pediatrics (84%), completed the survey. The majority (78%) received training on assessing ACEs and 60% reported using the Pediatric ACEs and Related Life-events Screener (PEARLS). About 22% “strongly agree” that PEARLS is acceptable and 32% “strongly agree” that PEARLS is feasible. Only 17% “strongly agree” that they like PEARLS. The top barriers were: (1) insufficient time; (2) unclear treatment pathway for detected ACEs; and (3) inadequate staffing to perform screening. The top facilitators for screening were: (1) financial incentives for providers to screen; (2) financial incentives for organizational leadership to implement screening; and (3) leadership support of screeners. The top approaches for addressing ACEs were: (1) behavioral therapy; (2) case navigation; and (3) trauma-informed care.

**Conclusion:**

This study provided a first look at provider perspectives on ACEs screening and treatment in a sample of California providers. Most responding providers report currently screening for ACEs and using PEARLS. Perceptions of feasibility were slightly higher than for acceptability. Facilitators were largely top-down and organizational in nature, such as financial incentives and leadership support. Future directions could include an exploration into why some providers may find ACEs unappealing and research to identify effective and accessible treatment approaches for ACEs.

## Introduction

Adverse childhood experiences (ACEs), a broad categorization referring to hardships and traumatic experiences experienced in early life, includes abuse (physical, emotional, and sexual), neglect (emotional and physical), household dysfunction (e.g., parental mental health and substance abuse problems), and social factors (e.g., poverty and food insecurity) (1–3). The impact of ACEs, when assessed across a population are associated with poor health and social outcomes and act in a dose–response manner, such that the greater number of ACEs, the more likely a person within a population will be experiencing toxic stress (chronic activation of the stress response systems) and related sequelae such as cardiovascular disease (4–6). The hypothesized reason for this impact is that ACEs lead to repeated activation of the sympathetic nervous system leading to overgeneralization of the stress response system and toxic stress (4–6). These stressors also lead to disruptions in neurologic, endocrine, immune, and metabolic systems with concomitant challenges in executive functioning (7). Adults who report four or more ACEs may be 2–2.3 times as likely to have a stroke, cancer, or heart disease, 3.1 times as likely to have chronic lower respiratory disease, 11.2 times as likely to have Alzheimer’s or dementia, and 37.5 times as likely to have attempted suicide compared to individuals within the population with no reported ACEs (8).

Despite growing evidence for ACEs as a risk factor for a variety of poor health outcomes, currently in the United States, clinical organizations rarely screen for ACEs as a preventative measure within the context of a primary care (9). However, beginning January 1, 2020, California became the first state to incentivize clinical organizations to introduce universal ACEs screening in primary care and identify patients at high risk of poor health outcomes (10). California incentivizes screening for all ages, birth to 69 years, and reimburses through Medi-Cal, the state’s safety net insurance provider (10). Between January 1, 2020 and June 30, 2022, Medi-Cal clinicians conducted more than 1,544,250 ACE screenings of 1,113,590 unique Medi-Cal members (8).

ACEs screening operates within a “screening-to-treatment” paradigm; by this, we mean an algorithm is used to screen patients, identify individuals with disease risk, and prescribe or refer to treatments matched to their risk profile. California State Board of Health (CSBH) adopted a specific screening instrument to promulgate ACEs screening, the Pediatric ACEs and Related Life-events Screener (PEARLS) developed by the Bay Area Research Consortium on Toxic Stress and Health (11). PEARLS encompasses three domains: abuse (physical, emotional, sexual), neglect (physical and emotional), and household dysfunction (parental incarceration, mental illness, substance abuse, parental separation, and intimate partner violence) (11). To promote PEARLS, CSBH launched an educational campaign known as “ACEs Aware” with a core training, “Becoming ACEs Aware in California” to certify clinicians to screen. The training is a free, two-hour webinar to learn about ACEs and ‘evidence-based’ care to effectively intervene on detected ACEs (10). Once clinicians are certified, they are eligible to receive $15 per PEARLS screening.

Given the recency of the ACEs Aware incentive policy within California, it is critical to examine early responses of clinicians in terms of perceived acceptability, feasibility and capacity for screening to be effectively integrated into the healthcare system. We conducted a survey during a three-month period from April to June 2022 among California clinicians (approximately 2 years after the launch of the policy) to explore provider knowledge about and perspectives on universal ACEs screening in clinical settings and the related intervention approaches to address detected ACEs. We hoped to glean initial insights on early indicators of acceptability and utility, as well as indicators of barriers and facilitators of screening during the initial launch and scaling of this approach. To our knowledge, this is the first study of provider perspectives after the roll-out of the ACEs Aware program.

### Objectives

O1: To explore the prevalence of ACEs screening across clinical organizations in California.
O2: To explore indicators such as acceptability, utility, barriers and facilitators of ACEs screening.

## Materials and methods

### Study design

This study employed a cross-sectional, quantitative and qualitative survey design to understand the landscape of ACEs screening, treatment, and implementation approaches in California approximately 2 years after the state incentivized ACEs screenings. We designed survey questions to evaluate implementation of screening in routine practice, provider perspectives on screening for ACEs, and treatment approaches. A team of researchers (*N* = 5) with expertise in implementation science, pediatrics, and behavioral science developed, reviewed, and revised survey questions to check for face validity. We built the survey in Qualtrics and did not alter questions after survey dissemination (Qualtrics, Provo, UT). We designed the survey to maximize user-friendliness and functionality based on recommendations for surveys (12). We offered an incentive of a raffle for a $100 Amazon gift card for one of the respondents chosen at random. The survey was exempt from IRB review (protocol #803048).

### Survey design

The anonymous survey was voluntary and distributed via Twitter, email, and Facebook using snowball sampling (13). The survey consisted of 26 items including multiple choice and free response questions to assess clinical background of the participants, determinants of ACEs screening, and treatment approaches for detected ACEs. We used a Likert scale from 1 (completely disagree) to 5 (completely agree) to measure acceptability and feasibility of PEARLS. The full survey is available in Supplementary file 1.

The first set of questions assessed background, role, and organizational experience of the respondent, including type and location of practice. Pediatricians on the study team developed the list of screening determinants based on their clinical expertise and research experience. Survey questions asked respondents to rank barriers or challenges with implementing ACEs screening with 1 = greatest barrier to screening and 8 = smallest barrier to screening. Survey questions asked respondents to use a drag-and-drop option to list the facilitators of screening in their preferred order from highest to lowest. Survey questions also asked respondents to rank interventions or treatment pathways for those with positive screens with 1 being most promising to 8 being least promising. To aid interpretation, we summarize the top three rankings in the results section and all rankings are available in Supplementary file 2.

### Acceptability and feasibility measures

The survey included the Acceptability of Intervention Measure (AIM) and Feasibility of Intervention Measure (FIM). The AIM and FIM demonstrated strong psychometric properties in a series of three studies conducted by Weiner et al. (14) with observed content validity, discriminant content validity, and reliability. These four-item measures of implementation outcomes are considered “leading indicators” of implementation success (15, 16). Cut-off scores for interpretation are not yet available; however, higher scores indicate greater acceptability and feasibility. The acceptability and feasibility of intervention measures were scored on a scale from 1 (completely disagree), 2 (disagree), 3 (neither agree nor disagree), 4 (agree), through 5 (completely agree). We computed mean scores for both measures and calculated the percent of participants selecting each response option. In this sample, reliability scores were excellent as measured by Cronbach’s Alpha, with *α* = 0.94 for the AIM and α = 0.93 for the FIM.

### Perspectives on evaluation

As an exploratory question, we asked participants to select which types of data that would be relevant to evaluate the value or impact of their ACEs screening program. The researcher team developed response options which included: improved child health, improved engagement in preventive care, improved attendance to preventive visits, decreased costs to organization, decreased costs to patients, and “other” with an open text box to describe other important metrics for program evaluation.

### Survey analyses

We collected and stored data in Qualtrics. Survey respondents provided consent electronically by selecting “Agree” or “Do Not Agree” to a standardized informational page before starting the survey (see Supplementary file 1) and their answers remained anonymous. We utilized the Checklist for Reporting Results of Internet E-Surveys (CHERRIES) to guide our survey reporting and analysis (12). Following the CHERRIES guidelines, we performed a completeness check of our survey data and removed responses with <25% completion rate or response times of <30 s which would suggest carelessness or fraudulent responses. Following the methodology used by Mlodzinski et al. (17), we also performed a quality analysis of the survey data and removed responses deemed suspicious for “bot” activity by flagging responses with exact matching free text responses or unintelligible responses. In total, 116 responses out of 198 were flagged and removed from the database (1 did not agree to participate, 4 included duplicate free-text responses, 15 for completing in <30 s, 16 were fully incomplete, and 80 had unintelligible free text responses). We used Excel to summarize data and calculate Chi-Square statistics for Table 1 (v.18.2110.13110.0).

**Table 1:** Characteristics of sample.

Organizational characteristics	*N* (%)	*p*-value^a^
Practice location (*n* = 82)		
California	82 (100%)	N/A
Practice type (*n* = 79)		0.000071
Private practice	28 (32%)	
Federally Qualified Health Center	22 (25%)	
Academic Medical Center	17 (21%)	
Community Health Center	12 (13%)	
Managed Care Organization	2 (2%)	
# of clinical practice sites (*n* = 80)		0.000102
10+	29 (36%)	
1	22 (28%)	
2	14 (18%)	
3–5	7 (9%)	
6–10	7 (9%)	
ACEs screening practices (*n* = 82)		3.50e−16
Currently screening	46 (56%)	
Not screening due to practice challenges or barriers	16 (20%)	
Not screening but would like to start	14 (17%)	
Not screening and not interested in starting	1 (1%)	
Other	4 (5%)	
ACEs screener (*n* = 40)		4.20e−07
PEARLS	36 (90%)	
Other	4 (10%)	
Provider characteristics		
Provider type (*n* = 84)		6.04e−15
Pediatrician (MD or DO)	39 (46%)	
Family Medicine Doctor (MD or DO)	23 (27%)	
Medical Assistant Family Medicine (MA)	4 (5%)	
Advanced Practice Family Medicine (NP)	3 (4%)	
Internist (MD or DO)	3 (4%)	
Received training on ACEs (e.g., using PEARLS) (*n* = 76)		2.07e−24
Yes	59 (78%)	
No	8 (10%)	
Not sure	7 (9%)	
Other	2 (3%)	

a *p-values are derived from Chi square tests.*

### Qualitative responses

We followed a pragmatic approach to qualitative data analysis with two researcher team members. This involved an open-coding process to categorize open-ended text responses to survey questions. All categories were created based on recurring key phrases, ideas, and words. From this initial grouping, emergent categories were “parent feedback,” “social workers,” “behavioral and mental health treatment for ACEs,” “screening facilitators,” and “ACEs screening for adults.” We discussed and drafted themes that encapsulated key ideas within each category. These themes were developed through an iterative process, where responses were continuously revisited. Themes were refined as data was reviewed. While we did not calculate inter-coder reliability (e.g., Cohen’s kappa), the analysis was iteratively conducted by two researcher team members. Throughout the coding process, both team members reviewed the codes regularly, resolving discrepancies through discussion until achieving 100% agreement. The final themes were reviewed and confirmed by the author team, ensuring consensus on identified themes. In future qualitative research, we plan to incorporate inter-coder reliability checks to enhance the rigor of the qualitative analysis and strengthen reliability.

## Results

### Sample characteristics and screening practices

Eighty two primary care providers completed demographic questions. Characteristics of the sample are listed in Table 1. All participants practiced in California (*n* = 82, 100%). The most common settings were private practice (*n* = 28, 35%), Federally Qualified Health Centers (FQHC) (*n* = 22, 28%), and academic medical centers (*n* = 17, 21%). Pediatricians (*n* = 39, 46%) and family medicine providers (*n* = 23, 27%) made up most participants. Most clinicians (*n* = 59, 78%) reported to have received training on ACEs screening, and around half (*n* = 46, 56%) reported that they are screening children for ACEs. Of the 46 providers who engaged in ACEs screening, 90% (*n* = 36) were using PEARLs screening tool. Missing data ranged from 7% to 11%.

### Acceptability and feasibility of PEARLS screening tool

Descriptive analyses for the AIM and FIM were limited to those who reported using the PEARLS screening tool (*n* = 35). Table 2 displays all acceptability and feasibility scores of PEARLS screening in primary care. The mean score on the AIM scale was 3.75/5 and the FIM was 4.0/5. On the 5-point Likert scale, the range for strongly agree was 29%–37% for questions asking if PEARLS is implementable, possible, doable, and easy to integrate. Similarly, the range for strongly agree was 17%–26% for questions asking if PEARLS meets their approval, is appealing, that they like PEARLS, or welcome PEARLS.

**Table 2:** Acceptability and feasibility of California PEARLS screening in primary care.

	Strongly agree	Somewhat agree	Agree	Somewhat disagree	Strongly disagree
	*n (%)*	*n (%)*	*n (%)*	*n (%)*	*n (%)*
Acceptability of intervention measure (*n* = 35)					
PEARLS meets my approval	9 (26)	17 (49)	7 (20)	1 (3)	1 (3)
PEARLS is appealing to me	9 (26)	13 (37)	8 (23)	4 (11)	1 (3)
I like PEARLS	6 (17)	16 (46)	10 (29)	3 (9)	0 (0)
I welcome PEARLS	7 (20)	15 (43)	9 (26)	2 (6)	2 (6)
Average (%)	22.25	43.75	24.5	7.25	3
Feasibility of intervention measure (*n* = 35)					
PEARLS is implementable	11 (31)	13 (37)	10 (29)	1 (3)	0 (0)
PEARLS is possible	10 (29)	18 (51)	7 (20)	0 (0)	0 (0)
PEARLS is doable	13 (37)	15 (43)	6 (17)	1 (3)	0 (0)
PEARLS is easy to integrate	11 (31)	9 (26)	10 (29)	4 (11)	0 (0)
Average (%)	32	39.25	23.75	4.25	0

	Mean	Standard deviation	Min	Max
Acceptability of intervention measure (*n* = 35)				
PEARLS meets my approval	3.91	0.91	1	5
PEARLS is appealing to me	3.71	1.06	1	5
I like PEARLS	3.71	0.85	2	5
I welcome PEARLS	3.66	1.04	1	5
Total mean	3.75			
Feasibility of intervention measure (*n* = 35)				
PEARLS is implementable	3.97	0.84	2	5
PEARLS is possible	4.09	0.69	3	5
PEARLS is doable	4.14	0.8	2	5
PEARLS is easy to integrate	3.79	1.02	2	5
Total mean	4	0.84	2.25	5

### Determinants of implementation of PEARLS

Table 3 lists the top three rankings for determinants of PEARLS implementation. The top three items identified as barriers to implementing PEARLS were “inadequate time,” “inadequate staffing to perform screener,” and “unclear treatment pathway for detected ACEs.” The top three items listed as facilitators for screening were “leadership support of screener,” “financial incentives for providers to screen,” and “financial incentives for organization.”

**Table 3:** Barriers, facilitators, and treatment approaches for adverse childhood experience (ACEs) in primary care.

	1st ranking, *n* (%)	2nd ranking *n* (%)	3rd ranking *n* (%)	Total votes
Facilitators to ACEs screening in primary care (*n* = 35)				
Leadership support of screener	8 (22%)	1 (3%)	15 (50%)	24
Financial incentives for providers to screen	10 (29%)	5 (14%)	4 (12%)	19
Financial incentives for organization to screen	2 (6%)	10 (29%)	5 (14%)	17
Staff knowledge of ACEs	6 (17%)	6 (17%)	3 (9%)	15
Additional time with patient provided	5 (14%)	5 (14%)	2 (6%)	12
Staff support of screener	0 (0%)	4 (12%)	4 (12%)	8
Staff trust in evidence behind screener	1 (3%)	2 (6%)	2 (6%)	5
Other facilitator [write-in]^a^	3 (9%)	2 (6%)	0 (0%)	5
Barriers to ACEs screening in primary care (*n* = 36)				
Inadequate time	20 (55%)	5 (14%)	5 (14%)	30
Inadequate staffing to perform screener	4 (11%)	18 (50%)	3 (8%)	25
Unclear treatment pathway for detected ACEs	7 (19%)	5 (14%)	5 (14%)	17
Lack of staff trust in screener	0 (0%)	4 (11%)	11 (31%)	15
Lack of staff awareness about screener	1 (3%)	2 (6%)	4 (11%)	7
Lack of staff knowledge of ACEs	0 (0%)	0 (0%)	4 (11%)	4
Lack of staff training in delivering screener	1 (3%)	1 (3%)	3 (8%)	5
Other barrier [write-in]^b^	2 (6%)	2 (6%)	0 (0%)	4
Promising treatments for disclosed ACEs (*n* = 21)				
Behavioral therapy	9 (43%)	2 (10%)	8 (38%)	19
Case navigators	6 (29%)	3 (14%)	5 (24%)	14
Trauma-informed primary care	3 (14%)	6 (29%)	3 (14%)	12
Community health workers	2 (10%)	5 (24%)	1 (5%)	8
Home visitation programs	0 (0%)	2 (10%)	3 (14%)	5
Group medical visits with a curriculum (e.g., Centering Parenting)	0 (0%)	1 (5%)	1 (5%)	2
Group parenting programs (e.g., Triple P, Incredible Years)	1 (5%)	1 (5%)	0 (0%)	2
Other [write-in]^c^	0 (0%)	1 (5%)	0 (0%)	1

a Other facilitators include “having provider champions.” All answers are in Supplementary file 2.

b Other barriers include “parents not understanding how to fill out screener.” All answers are in Supplementary file 2.

c Other promising treatments include “engage with smart care to work on resiliency.” All answers are in Supplementary file 2.

### Qualitative perspectives on ACE screening and treatment

Table 4 lists themes that emerged through open-text responses with exemplary quotes. ACEs screening seemed challenged by lack of resources and services for parents. Providers noted that there needed to be more educational (“more parenting tools”) and counseling resources (“I wish we had a social worker to help with housing,” “more behavioral health support for children”) for parents. Providers shared that ACE screening could be expanded to adult populations, “Every problem I was seeing in my adult patients was rooted in ACEs.” Results suggest that screening for ACEs is perceived as feasible with the appropriate staff and collaborations. Example facilitators were, “collaboration between family physicians and our case managers and behavioral specialists” and “having provider champions.”

**Table 4:** Qualitative themes on adverse childhood experience screening and treatment.

Qualitative themes	Quote 1	Quote 2	Quote 3
*Need for more parental support resources*	*“Parents not understanding how to fill out questionnaire”*	*“Parent not understanding who should answer”*	*“All answers written on paper say no, parent open to talk about them in exam room”*
*ACEs screening manageable with the appropriate staff and processes in place*	*“Resources identified”*	*“Having provider champions”*	*“Having referral process for positive screens”*
*More available behavioral/mental health treatment for ACEs*	*“We are considering a behavioral health specialist on site”*	*“I wish there were more available mental health providers”*	*“More behavioral health support for children”*
*Implementing routine ACEs screening with adults*	*“Too many [college] students have high ACEs scores and need the help they could not get when they were younger”*	*“We need to implement ACEs screening for adults”*	*“Every problem I was seeing in my adult patients was rooted in ACEs”*
*Importance placed on the presence of a social worker*	*“Emergency social worker in office”*	*“Social worker to find community resources for basic needs”*	*“I wish we had a social worker to help with housing”*

### Treatments and interventions for ACEs

Table 3 displays the rank-ordered most promising treatments for ACEs and Table 5 displays current routine care practices. Most providers who reported screening agreed (*n* = 32, 89%) that the first step when a child screens positive for ACEs was to assess or treat the child’s health conditions related to ACEs (e.g., anxiety, ADHD, obesity). Family education on self-care activities (e.g., nutrition, sleep, stress management) (*n* = 30, 83%) and the connection between ACEs and associated future risks (*n* = 29, 81%) were also recognized as current intervention options for identified ACEs. Behavioral therapy (e.g., parent–child interaction therapy) (*n* = 9, 43%), case navigation (*n* = 6, 29%), and trauma-informed primary care (*n* = 3, 14%) were the most promising treatment approaches for disclosed ACEs.

**Table 5:** Routine care pathways for adverse childhood experiences screening.

If child screens positive for ACEs, what most commonly happens next? (*n* = 36)	*n* (%)
Assess/treat child health condition	32 (89%)
Educate family about self-care	30 (83%)
Educate family about ACEs and associated risks	29 (81%)
Refer to behavioral health provider for evidence-based practice (e.g., parent child interaction therapy, cognitive behavioral therapy, or trauma focused psychotherapy)	27 (75%)
Assess child self-regulation behavior	18 (50%)
Assess and refer for social needs	17 (47%)
Refer to healthy developmental services or baby steps	15 (42%)
Assess parenting or co-regulatory behaviors	13 (36%)
Refer to existing evidence-based parenting programs	9 (25%)
Refer to group visits or shared medical appointments	7 (19%)
Recruit on-site parenting programs	6 (17%)
Other treatments or interventions	4 (11%)
Refer to home visiting programs	4 (11%)

### Determinants of ACEs screening by practice type

In federally qualified health centers and community health centers (FQHCs), 23 providers (70%) reported screening for ACEs, 6 (18%) would like to start, 5 (15%) cited practice-related barriers, and 4 (12%) provided other reasons. The most frequently reported barriers were inadequate time (*n* = 13) and unclear treatment pathway for detected ACEs (*n* = 4). For facilitators, the highest-ranked factors were leadership support of the screener (*n* = 6), financial incentives for providers to screen (*n* = 6), and staff knowledge of ACEs (*n* = 3). In private practice, 20 providers (71%) reported screening for ACEs, 6 (21%) would like to start, 1 (4%) faced practice challenges, 1 (4%) was not interested in starting, and 1 (4%) cited other reasons. The most frequently reported barriers were inadequate time (*n* = 6), inadequate staffing (*n* = 3), and unclear treatment pathways (*n* = 3). For facilitators, the top-ranked were leadership support of the screener (*n* = 3), additional time with patients (*n* = 3), and staff knowledge of the screener (*n* = 3). In academic medical centers and managed care organizations, 6 providers (35%) reported screening for ACEs, 2 (12%) would like to start, 9 (53%) cited practice-related barriers, and 2 (12%) provided other reasons. For barriers, 13 (100%) ranked inadequate time as the highest barrier, and 4 (31%) ranked unclear treatment pathways as the next highest. The top facilitators included financial incentives (*n* = 6), leadership support (*n* = 6), and staff knowledge of ACEs (*n* = 3).

## Discussion

Although ACEs have garnered widespread media attention for influencing health, a robust approach for addressing them is still not commonplace across the United States. California is the first state to enact a policy to incentivize ACEs screening in primary care with the launch of ACEs Aware in January 2021, as one possible strategy for meaningfully addressing the deleterious effects of ACEs. This presents a unique opportunity to explore perspectives of clinicians administering screenings to understand the extent to which ACEs screening is acceptable, feasible, fits into the workflow of primary care, and appears to be working as intended toward reducing the deleterious effects of ACEs. To our knowledge, this is the first study to evaluate perspectives of California providers conducting ACEs screening during the first few years of the California roll-out.

In this study, we found that more than half of respondents were screening children for ACEs in their primary care practices at the time of the study (April–May 2022) and most clinicians reported receiving training on ACEs screening. This diverges from other cross-sectional studies on ACEs which found that clinicians have limited familiarity with the effects of ACEs, few received training on ACEs, and only some formally screen their patients for ACEs (18). However, these studies may not be directly comparable given our self-selecting sample which may have introduced reporting bias.

Although ACEs screening was relatively common in this sample, barriers emerged such as insufficient time and lack of staffing to support screening. Other themes emerged characterizing on-the-ground barriers including lack of robust structures and systems to support the influx of positive screens as well as lack of a clear set of decision-rules for guiding the match from screening to treatment options. This is consistent with an ACEs Aware report underscoring that lack of time, limited clinical infrastructure, and shortage of skilled support staff were leading obstacles in adopting ACEs screening (19). Although perceived time constraints are a common barrier (20), ACEs screening was found to impact time only minimally (21) and increase visit length by about 5 min (22). Providers’ perceptions about lack of time may be indicative of underlying questions about their role as a primary care provider and whether ACEs screening fits in the scope of a conventional primary care visit. It is noteworthy that feasibility scores were higher than that of acceptability (4/5 vs. 3.75/5), indicating that screening may be feasibly integrated, but acceptability and openness to screening (on the part of the providers) may be lower. This aligns with recent provider critiques of ACEs screening about increased stigma for already marginalized groups and the potential to generate undue parental blame (23).

Facilitators of screening were financial and organizational in nature. Monetary incentives for both providers and the organization were highly rated facilitators. This is expected given that reimbursement rates for well-child visits are historically low and health systems are underfunded (2). However, in practice, the impact of financial incentives on provider screening behaviors is typically minimal and heterogeneous (24). These financial incentive systems could also be construed as coercive, implying that organizations and providers only implement ACEs screening (or think that it is necessary) with external fiscal pressures. Leadership support is another highly rated facilitator, with extant research demonstrating that effective and supportive leadership is foundational for organizational change (25). With that said, leadership pressure could also be perceived as coercive if done in a way that mandates the use of screening instead of educates and supports cultural adoption of it.

Across practice types, private practices and community health centers reported higher screening rates (71% and 70%, respectively), while academic medical centers and managed care organizations had a lower rate (35%). These differences may reflect the flexibility of smaller practices versus more complex structures in larger organizations. Overall, barriers to ACEs screening are common across all practice types, with inadequate time being the most significant. Facilitators like leadership support and staff knowledge were universally recognized as essential. However, the small sample sizes across groups limit the ability to interpret these findings, and further research with larger cohorts is needed to draw more definitive conclusions.

Providers reported that the most common procedure when a child screens positive for ACEs is to assess or treat the child’s related condition such as anxiety, ADHD, or obesity. This illuminates questions about the use of a screen-to-treat paradigm in relation to a concept as complex as ACEs. For some who are resistant to screening, it is unclear what the added value of ACEs screening is given that the treatments are often offered during other screening and assessment processes. Providers also rated family self-care and parenting education as common offerings for ACEs that could be universally appropriate and provided regardless of score. Thus, it is unclear what added value the ACEs screening approach, as currently implemented, does for improving care. Future work could focus on identifying exactly what an appropriate response should be for positive screens, with attention to understanding the added value of ACEs for identifying latent risk beyond other focused clinical and developmental screenings. To that end, one of the other common solutions to a positive ACEs screen was to refer to a behavioral health practitioner, consistent with other work (1). Our qualitative findings also reiterate that having mental health professionals, such as psychologists or social workers, on patient care teams is critical for timely follow-up and patient engagement. While this makes sense within the context of a screen-to-treat paradigm, issues may arise as prior work suggests that patients may experience behavioral referrals as unnecessary and potentially unhelpful (26). While further research is needed, to illustrate options, an alternative approach could be to provide support universally related to toxic stress and encourage all families to integrate stress-relieving activities and self-care into daily life.

ACEs screening remains controversial with critical questions around stigma, medical surveillance, discrimination, and the exacerbation of medical mistrust. Leading organizations such as American College of Preventive Medicine have opposed screening for ACEs within individual encounters (27). Critics emphasize ACEs screening is predictive of increased health risk across a population, but when examined across time for individuals, ACEs does not predict future deleterious health outcomes (28). There is also heterogeneity in patient disclosure of ACEs; one study found between 6% to 64% reported one or more ACEs and 0.01% to 40.7% reported four or more ACEs (29). This may be due to inconsistent reporting given stigma and sensitivity of discussing adversity within a short visit. Taken together, the results from this study in the context of related work suggest that although ACEs screening is feasible, it is relatively less acceptable to providers and there is inconsistency around treatment approaches to ACEs in primary care. We posit a primary reason for ACEs being adopted in California is a pathway for billability by healthcare organizations. ACEs screening has become increasingly institutionalized without attention to whether screening is acceptable and whether screening leads to appropriate, feasible, and impactful health outcomes above current well child visit practice.

Alternatives to the screen-to-treat paradigm to ACEs have been suggested before, such as the Healthy Outcomes through Positive Experiences (HOPE^®^) Framework (30). HOPE^®^ offers a paradigm shift from a screen-to-treat, problem-solving approach in pediatric care to, instead, an approach that focuses on assets, strengths, and relationship cultivation (30). There is recognition of adversity, but there is also an emphasis on family strengths as a buffering effect to adversity (31). Prior work found that positive childhood experiences (PCE), such as having a supportive caregiver and family, can moderate the effects observed by ACEs across populations (31). So much so that, in the context of a score of 6 out of 6 on the PCE, there is limited to no impact on a range of health outcomes from high scores on ACEs screens (31). While speculative, a strengths-based approach focused on cultivating PCEs, with appropriate training and support for pediatricians, might be met with less ambivalence by providers and families and thereby increased adoption in a more bottom-up way.

### Strengths and limitations

Our survey, distributed via Twitter, email listservs, and Facebook, reached primary care providers across California, supporting a cost-effective approach to recruitment. We acknowledge that the sample may have been biased and was predominantly from Southern California; and the professional networks like the American Academy of Pediatrics San Diego Chapter and the Children’s Primary Care Medical Group (CPCMG) may have influenced participation. Although our respondents span between Sacramento, CA and San Diego, CA, the findings may not be representative of all California providers. Future research should aim for more geographic diversity, particularly from Northern California, and include additional demographic data to better understand provider experiences across localities.

Our study does have several strengths including employing validated measures (i.e., AIM and FIM), recruiting a sample of providers based in California with experience with the ACEs Aware roll-out, and using both quantitative and qualitative measures to contextualize findings. Despite these strengths, our approach has notable limitations. First, despite using multiple forms of data collection, due to privacy issues, we did not collect demographic or other identifying information. Thus, we are unable to draw conclusions about subgroups (e.g., based on gender or race) or perform analyses to address confounders. Second, we do not know the view rate for the survey, which may have helped us to understand study reach. Third, as with any survey, there are risks of both selection bias as well as participation bias. With an online-only survey, it may not have captured eligible participants who do not engage in social media or the internet regularly. Prospective eligible participants could not have participated for various reasons, such as being uninterested in research, or the subject matter itself. Snowball sampling was used to address this limitation, as this approach helped to recruit interested participants by word-of-mouth while utilizing the professional network of those involved. However, snowball sampling carries a risk of inherent biases. Fourth, analyzing the quantitative and qualitative data independently within the survey study allowed for complementary insights, but did not enable direct cross-validation between the two data sources. Future research can integrate qualitative and quantitative findings to strengthen the study’s conclusions.

However, from available data, our findings appear consistent with existing reports on California-based ACEs screening (8). Fifth, the truly open nature of the social media survey led to unanticipated “bot” responses, and while steps were taken to remove suspicious responses, there is no validated or definitive means to filter bot activity. Despite these limitations, we view our findings as an important first step in understanding the novel ACEs screening policy in primary care.

## Conclusion

Despite the promise that universal ACEs screening in primary care can save lives, critical questions around the utility of ACEs screening emerged from this study. Although, we found that most California providers in this sample agree that ACEs screening is feasible, it is noteworthy that acceptability was slightly lower. This may be, in part, related to the fact that positive ACE findings do not seem to result in well-specified next actions within primary care. We found an inconsistency in treatment pathways and lack of resources to support children and families who screen positive for ACEs. This study revealed several potential areas for future research. First, there are opportunities for further investigation including why ACEs screening may be less acceptable and/or less appropriate in certain contexts. There are opportunities to explore how and if ACEs screening influences care beyond traditional well child visit models and whether there are medium- and long-term impacts to addressing ACEs early. Another future area of work could involve identifying effective treatments and optimizing care pathways for families and children with ACEs, to improve the process of addressing adversity in primary care. Finally, there is opportunity to explore alternatives to a problem-solving oriented screen-to-treat paradigm in relation to ACEs and, instead, shift toward a model such as HOPE^®^, which focuses on promoting PCEs for everyone as a buffer against the deleterious health impacts of ACEs.

## Data Availability

The raw data supporting the conclusions of this article will be made available by the authors, without undue reservation.
