# Supplementary material for: The California adverse childhood experiences screening roll-out: a survey study of ACEs screening implementation in primary care
Source: Front Public Health. 2025 Apr 2;13:1446555. doi: 10.3389/fpubh.2025.1446555 (PMC12000056; doi:10.3389/fpubh.2025.1446555)
Supplement: Supplementary file 1 [file Supplementary_file_1.pdf]

UC San Diego Health

AGREEMENT

Pediatric primary care providers' experiences addressing Adverse Childhood Experiences in practice

You are being invited to participate in a project titled "Pediatric primary care providers' experiences addressing Adverse Childhood Experiences (ACEs) in practice". This study is being led by Eric Hekler, Clare Viglione, Gregory Aarons, Pradeep Gidwani and Blanca Melendrez from University of California San Diego to understand provider practices and perspectives regarding ACE screening in primary care as well as the associated treatment practices.

Data from this project will be used to describe the landscape of Adverse Childhood Experiences (ACEs) screening in pediatric primary care as well as the associated treatment practices. Our objective is to include those involved in the practice of primary care.

This anonymous survey should take 5-10 minutes to complete. Your participation is entirely voluntary and you can withdraw at any time by simply exiting. All data collected

up to the point of exiting the survey will be retained. If you wish to have all of your data removed, please contact cviglione@health.ucsd.edu. You are also free to skip any question that you choose. You have the option to provide your email address at the end of the survey for a chance to win a prize. Your chances are approximately 1 in 200.

If you want additional information or have questions, you may contact cviglione@health.ucsd.edu. Thank you so much!

By clicking "I agree", you are agreeing to proceed to the survey and share data with the study team about your experience addressing ACEs in pediatric primary care.

- I Agree
- I Do Not Agree

BACKGROUND

Thank you for agreeing to complete this survey about your experiences in pediatric primary care! This survey will help us learn more about the best ways to address Adverse Childhood Experiences (ACEs) and improve child health and development.

Captcha verification:

☐ I'm not a robot reCAPTCHA Privacy - Terms

Do you practice pediatric primary care, family or internal medicine in which you provide primary health care to children?

- ○ Yes
- ○ No

Do you practice or work in California?

- ○ Yes
- ○ No
- ○ [ ] Other Location

Select your role(s)

- ☐ Pediatrician (MD or DO)
- ☐ Internist (MD or DO)

- ☐ Family medicine doctor (MD or DO)
- ☐ Advanced Practitioner in pediatrics (NP)
- ☐ Advanced Practitioner in family medicine (NP)
- ☐ Nurse in pediatrics (RN, LVN, etc.)
- ☐ Nurse in family medicine (RN, LVN, etc.)
- ☐ Behavioral health clinician (MSW, LCSW, MFT, etc.)
- ☐ Medical Assistant in pediatrics (MA)
- ☐ Medical Assistant in family medicine (MA)
- ☐ Urgent care provider
- ☐ Emergency care provider
- ☐ Other: ____________

Which age groups do you treat?

- ☐ 0-5
- ☐ 5-10
- ☐ 10-16
- ☐ 16-18
- ☐ 18-21

How would you describe your clinical or practice setting?

- ☐ Private practice
- ☐ Academic Medical Center

☐ Managed Care Organization (e.g., Kaiser Permanente)

☐ Federally Qualified Health Center

☐ Community Health Center

☐ Military Clinic

☐ Free Clinic

☐ Other: ________

Optional: Name of your current clinic or organization

____

How many clinical practice sites does your organization have?

○ 1

○ 2

○ 3-5

○ 6-10

○ 10+

Have you received training or education on assessing Adverse Childhood Experiences and related life events in

primary care (e.g., PEARLS)?

- Yes
- No
- Not sure
- Other:

SCREENING

Are you and/or your care team currently screening children for their exposure to ACEs? Select the response(s) that best describe your current practice. Select all that apply.

- Yes
- Not screening but we would like to start
- Not screening due to practice challenges/barriers
- Not currently screening and not interested in starting
- Other:

Enter the name of the ACEs screener you are currently using

- Pediatric ACEs and Related Life Events Screener (PEARLS)

○ Other: [ ]

Rank the barriers or challenges with implementing ACEs screening in your practice **from #1 (greatest barrier to screening) to #8 (smallest barrier to screening).**

Drag and drop the barriers to list in your preferred order.

Optional: You may write-in an additional barrier in "Other" and include in the ranking.

Inadequate time

Inadequate staffing to perform screener

Lack of staff trust in screener

Lack of staff awareness about screener

Lack of staff knowledge of ACEs

Lack of staff training in delivering screener

Unclear treatment pathways for detected ACEs

Other barrier: [ ]

Rank the factors associated with *improved* delivery of ACEs screening **from #1 (most helpful factor) to #8 (least helpful factor).**

Drag and drop the statements to list in your preferred order.

Optional: You may write-in an additional factor in "Other" and include in the ranking.

Financial incentives for provider to screen

Financial incentives for organization to screen

Leadership support of screener

Staff support of screener

Staff trust in evidence behind screener

Additional time with patient provided

Staff knowledge of ACEs

Other factor: [ ]

Rate your agreement with the following statements about the implementation of Pediatric ACEs and Related Life Events Screener (PEARLS) in routine practice.

	Completely disagree (1)	Disagree (2)	Neither agree nor disagree (3)	Agree (4)	Completely agree (5)
PEARLS meets my approval.	○	○	○	○	○
PEARLS is appealing to me.	○	○	○	○	○
I like PEARLS.	○	○	○	○	○
I welcome PEARLS.	○	○	○	○	○
PEARLS is implementable.	○	○	○	○	○
PEARLS is possible.	○	○	○	○	○
PEARLS is doable.	○	○	○	○	○
PEARLS is easy to integrate	○	○	○	○	○

These questions will help us understand current treatments to address childhood ACEs and issues with self-regulation. Please read the questions carefully.

If children screen positive for ACEs, what do providers at your clinic **typically offer** or do as the next step?

Select all *routine* treatment options available at your clinic and where possible, enter the specific name of the therapy or intervention.

- ☐ Educate families on importance of ACEs, future risks, and/or toxic stress
- ☐ Educate families about self-care (e.g., nutrition, sleep, stress management)
- ☐ Assess parenting or co-regulation behaviors (e.g., caregiver's ability to interpret and respond to child cues, ability to calm child, family structure and routines)
- ☐ Assess and treat child health conditions related to ACEs (e.g., anxiety, ADHD, obesity, etc.)
- ☐ Assess child self-regulation behaviors (e.g., regulating emotions, following directions, focusing on specific tasks, waiting turns, or resisting eating unhealthy food)
- ☐ Refer to behavioral health provider for evidence-based therapy (i.e., parent child interaction therapy, cognitive behavioral therapy, or trauma focused psychotherapy)

  [ ]
- ☐ Refer to external evidence-based parenting programs (e.g., Incredible Years, Triple P, etc.)

  [ ]

- [ ] Refer to home visiting programs such as (e.g., Nurse Family Partnership, SafeCare, etc.)
- [ ] Recruit for on-site parenting program
- [ ] Refer to group visit or shared medical appointments (e.g., CenteringParenting)
- [ ] Refer to Healthy Development Services or Babysteps
- [ ] Assess and refer for social needs (e.g., housing, Calfresh (SNAP), etc.)
- [ ] Other treatment or intervention:

Optional: Please elaborate on the answer options selected in the previous question and/or provide additional context about your ACEs treatment workflow.

SELF REGULATION

Are you or your care team currently screening children for challenges with self-regulation such as issues with controlling emotions, following directions, focusing on specific tasks, or waiting turns? Select all that apply.

- ☐ Yes
- ☐ Not screening but we're considering starting
- ☐ Not screening because of practice challenges or barriers
- ☐ Not currently screening and not interested in starting
- ☐ [ ] Other:

Enter the name of the behavioral screener you are currently using and/or briefly describe the process of assessing child self-regulation in primary care:

[ ]

If children present with self-regulation issues, what do providers at your clinic typically offer or do as the next

step?

Select all routine treatment options available at your clinic and if possible, enter the name of each therapy or intervention.

- ☐ Educate families about importance of child self-regulation for optimal development
- ☐ Educate families about self-care activities (e.g., nutrition, sleep, activity)
- ☐ Assess parenting or co-regulation behaviors (e.g., caregiver's ability to interpret and respond to child cues, ability to calm child, family structure and routines)
- ☐ Clarify specific behavioral challenges and then tailor feedback
- ☐ Refer to behavioral health provider for evidence-based therapy (i.e., parent child interaction therapy, cognitive behavioral therapy, or trauma focused psychotherapy) ________
- ☐ Refer to external evidence-based parenting programs (e.g., Incredible Years, Triple P, etc.) ________
- ☐ Refer to home visiting program such as (e.g., Nurse Family Partnership, SafeCare, etc.) ________
- ☐ ________ Recruit for on-site parenting program
- ☐ Refer to group visit or shared medical appointments with a curriculum (e.g., CenteringParenting) ________

☐ Assess and refer for social needs (e.g., housing, Calfresh (SNAP), etc.)

☐ Other:

What ACEs treatment options do you wish were available for referral or intervention at your clinic?

Rank the following interventions or approaches to address developmental or behavioral challenges related to ACEs **from #1 most promising to #8 least promising**.

Drag and drop the interventions to list in your preferred order.

Optional: You may write-in an additional intervention in "Other" and include in the ranking.

behavioral therapy (e.g., parent-child interaction therapy)

community health workers

case navigators

trauma-informed primary care

home visitation programs

group medical visits with a curriculum (e.g., CenteringParenting)

group parenting programs (e.g., Triple P, Incredible Years)

Other:

PREPARATION

OPTIONAL FINAL QUESTIONS: The final set of questions are about curriculum-based, group medical visits such as CenteringPregnancy or CenteringParenting. Group visits with families are a promising approach to address toxic stress and child developmental issues.

In considering the implementation of group visits at your practice, rank the following barriers **from #1 (greatest potential barrier to implementation) to #10 (least likely to be a barrier to implementation).**

Drag and drop the barriers to list in your preferred order.

Optional: You may write-in an additional barrier in "Other" and include in the ranking.

Inadequate time

Inadequate staffing

Lack of provider trust

Lack of provider knowledge

Lack of leadership support

Too expensive

Negative provider attitudes

Inability to recruit families for group visits

Lack of attendance to group visits

Other: [ ]

What data do you think would be **most important to your organization to evaluate *program success*?**

- ☐ Increased attendance to preventive visits
- ☐ Improved engagement in preventive care (e.g., immunizations)
- ☐ Improved child health
- ☐ Decreased costs to organization
- ☐ Decreased costs to patients
- ☐ [ ] Other:

END

Anything else that you would like the UC San Diego study team to know?

[ ]

If you would like to be entered into a raffle to earn a $100 Visa giftcard, please enter your email.

Powered by Qualtrics
