# Supplementary material for: The California adverse childhood experiences screening roll-out: a survey study of ACEs screening implementation in primary care
Source: Front Public Health. 2025 Apr 2;13:1446555. doi: 10.3389/fpubh.2025.1446555 (PMC12000056; doi:10.3389/fpubh.2025.1446555)
Supplement: Supplementary file 2 [file Supplementary_file_2.docx]

| **Supplemental File 2 – Barriers, Facilitators, and Treatment Approaches for ACEs – All Rankings** | | | | | | | | | | |
| --- | --- | --- | --- | --- | --- | --- | --- | --- | --- | --- |
| **Ranked Barriers to ACEs** | | | n=36 |  |  |  |  |  |  |  |
|  |  |  | **1** | **2** | **3** | **4** | **5** | **6** | **7** | **8** |
| Inadequate time | |  | 20 | 5 | 6 | 2 | 0 | 1 | 1 | 1 |
| Inadequate staffing to perform screener | | | 4 | 18 | 3 | 5 | 3 | 2 | 0 | 1 |
| Lack of staff training in delivering screener | | | 1 | 1 | 3 | 3 | 8 | 13 | 5 | 2 |
| Lack of staff knowledge of ACEs | | | 0 | 0 | 4 | 7 | 12 | 8 | 4 | 1 |
| Unclear treatment pathways for detected ACEs | | | 7 | 5 | 5 | 3 | 2 | 2 | 12 | 0 |
| Lack of staff trust in screener | | | 0 | 4 | 11 | 6 | 1 | 6 | 7 | 1 |
| Lack of staff awareness about screener | | | 1 | 2 | 4 | 10 | 9 | 4 | 7 | 0 |
|  |  |  |  |  |  |  |  |  |  |  |
| **Other Barrier** | |  |  |  |  |  |  |  |  |  |
| Text: |  |  |  |  |  |  |  |  |  |  |
| n/a |  |  |  |  |  |  |  |  |  |  |
| Access to couns/psych services, brief psychotherapy | | | |  |  |  |  |  |  |  |
| Parents not understanding how to fill out questionnaire | | | |  |  |  |  |  |  |  |
| Responses on paper say no, but willing to discuss in exam room | | | | |  |  |  |  |  |  |
| No barriers, ACEs fully integrated in workplace | | | |  |  |  |  |  |  |  |
| Parent not understanding who should answer | | | |  |  |  |  |  |  |  |
| Lack of reimbursement for time | | |  |  |  |  |  |  |  |  |
| Overcoming other screening priorities | | |  |  |  |  |  |  |  |  |
|  |  |  |  |  |  |  |  |  |  |  |
|  |  |  |  |  |  |  |  |  |  |  |
|  |  |  |  |  |  |  |  |  |  |  |
| **Facilitators Ranked to ACEs** | | | | n=35 |  |  |  |  |  |  |
|  |  |  | **1** | **2** | **3** | **4** | **5** | **6** | **7** | **8** |
| Finan. incent. prov. to scr | |  | 10 | 5 | 4 | 3 | 1 | 4 | 6 | 2 |
| Finan. incent. org. to scr | |  | 2 | 10 | 5 | 3 | 6 | 4 | 3 | 2 |
| Ldrshp support of Scr. | |  | 8 | 1 | 15 | 2 | 3 | 3 | 2 | 1 |
| Staff support of screener | |  | 0 | 4 | 4 | 17 | 4 | 5 | 1 | 0 |
| Staff trust in evidence behind screener | | | 1 | 2 | 2 | 2 | 14 | 6 | 8 | 0 |
| Additional time with patient provided | | | 5 | 5 | 2 | 4 | 4 | 11 | 2 | 2 |
| Staff knowledge of ACEs | |  | 6 | 6 | 3 | 4 | 3 | 1 | 11 | 1 |
| Other factor |  |  | 3 | 2 | 0 | 0 | 0 | 1 | 2 | 27 |
|  |  |  |  |  |  |  |  |  |  |  |
|  |  |  |  |  |  |  |  |  |  |  |
| **Other Facilitator** | |  |  |  |  |  |  |  |  |  |
| Text: |  |  |  |  |  |  |  |  |  |  |
| Useful clinically | |  |  |  |  |  |  |  |  |  |
| Collab. btwn family physicians/case managers/behav.health special, and CAPS | | | | | |  |  |  |  |  |
| Patient benefits of screening | | |  |  |  |  |  |  |  |  |
| Resources identified | |  |  |  |  |  |  |  |  |  |
| Having it built in, and all other questions upfront | | | |  |  |  |  |  |  |  |
| Having referral process for positive screens | | | |  |  |  |  |  |  |  |
| Having provider champions | |  |  |  |  |  |  |  |  |  |
|  |  |  |  |  |  |  |  |  |  |  |
|  |  |  |  |  |  |  |  |  |  |  |
|  |  |  |  |  |  |  |  |  |  |  |
| **Routine treatment pathways for ACEs** | | | | |  |  |  |  |  |  |
|  |  |  |  |  |  |  |  |  |  |  |
|  |  |  |  |  |  |  |  |  |  |  |
| **Approaches & Treatments to ACEs - Ranked** | | | | n=21 |  |  |  |  |  |  |
|  |  | **1** | **2** | **3** | **4** | **5** | **6** | **7** | **8** |  |
| Oth. |  | 0 | 1 | 0 | 0 | 0 | 0 | 0 | 20 |  |
| Behav. ther. (par/chld) | | 9 | 2 | 8 | 0 | 1 | 1 | 0 | 0 |  |
| Grp. parent. prgms. | | 1 | 1 | 0 | 5 | 3 | 2 | 9 | 0 |  |
| Grp. med. visits w/curr. | | 0 | 1 | 1 | 2 | 1 | 8 | 8 | 0 |  |
| Comm. hlth. w. | | 2 | 5 | 1 | 3 | 7 | 3 | 0 | 0 |  |
| Case nav. |  | 6 | 3 | 5 | 2 | 1 | 3 | 1 | 0 |  |
| Home visit. prgms. | | 0 | 2 | 3 | 5 | 6 | 2 | 3 | 0 |  |
| TI Prim. care |  | 3 | 6 | 3 | 4 | 2 | 2 | 0 | 1 |  |
|  |  |  |  |  |  |  |  |  |  |  |
